# An overview of hereditary spherocytosis and the curative effects of splenectomy

**DOI:** 10.3389/fphys.2025.1497588

**Published:** 2025-02-11

**Authors:** Kyril Turpaev, Elizaveta Bovt, Soslan Shakhidzhanov, Elena Sinauridze, Nataliya Smetanina, Larisa Koleva, Nikita Kushnir, Anna Suvorova, Fazoil Ataullakhanov

**Affiliations:** ^1^ Center for Theoretical Problems of Physicochemical Pharmacology, Russian Academy of Sciences, Moscow, Russia; ^2^ Dmitry Rogachev National Medical Research Center of Pediatric Hematology, Oncology and Immunology, Moscow, Russia; ^3^ Moscow Institute of Physics and Technology, National Research University, Dolgoprudny, Russia; ^4^ Perelman School of Medicine, University of Pennsylvania, Philadelphia, PA, United States

**Keywords:** hereditary spherocytosis, gene mutations, erythrocyte cytoskeleton, erythrocyte deformability, spherocytes, microcirculation, interendothelial slits, splenectomy

## Abstract

Hereditary spherocytosis is a common hemolytic anemia with different severity. The causes of hereditary spherocytosis are mutations in genes that encode red blood cell (RBC) membrane and cytoskeletal proteins, including ankyrin-1, Band 3 (or AE1), α spectrin, β spectrin, and protein 4.2. Molecular defects in these proteins decrease membrane integrity, leading to vesiculation, decreased membrane surface area, and reduced deformability of the cells. Eventually, this leads to the trapping the abnormal RBCs (spherocytes) in the spleen. In most severe cases, splenectomy may be necessary to prevent general RBC collapse during the passage of RBCs through the narrow slits of venous sinuses in the spleen. The clinical benefit of splenectomy results from elimination the primary site of RBC damage and destruction. Splenectomy is a curative approach but can cause complications and should be undertaken after examination by various laboratory approaches. Splenectomy does not correct most genetically determined membrane abnormalities in erythrocytes in patients with hereditary spherocytosis. The transformation of biconcave erythrocytes into spherocytes continues, although to a lesser degree than before surgery. Nevertheless, splenectomy increases the lifespan of red cells, significantly reducing the severity of anemia and improving many physiological signs of HS.

## 1 Introduction

Hereditary spherocytosis (HS), also known as Minkowski Chauffard disease or spherocytic anemia, was first identified and characterized at the end of the 19th century by the presence of abnormally shaped spherical erythrocytes (spherocytes) in blood samples. HS, along with its weakly expressed subclinical forms, is one of the most common types of hemolytic anemias. Among the most typical features of HS are anemia, chronic hemolysis accompanied by an increase in unconjugated bilirubin and decrease in haptoglobin levels, splenomegaly, jaundice, and gallstones. Of the characterized cases, 75% are mediated by autosomal dominant inheritance (disease was manifested in previous generations), while approximately 25% of cases are due to either recessive or new mutations. In European countries and North America, the incidence of spherocytosis is about 1 in 2000–5000 people, in China it is about 1 in 70,000 people (Da Costa et al., 2013; Delaunay, 2007; Eber and Lux, 2004; Seregina et al., 2019; Perrotta et al., 2008; Kalfa, 2021).

## 2 The life cycle of red blood cells

The generation of red blood cells (RBCs) occurs in the bone marrow hematopoietic compartments. The precursors of erythrocytes are erythroblasts, which are produced by differentiation of less mature precursors (erythroid progenitors). Erythropoietin and other factors induce the differentiation of precursors into erythroblasts and activate the transcription of the globin gene and hemoglobin accumulation. Erythropoietin is a peptide hormone whose production by the kidneys is induced under hypoxic conditions. During the process of differentiation of erythrocyte precursors, the expression of the erythropoietin receptor and the rate of proliferation gradually decrease. At the stage of orthochromatic erythroblasts, the cells undergo enucleation, transforming into reticulocytes. To leave the bone marrow and enter the bloodstream, the newly generated reticulocytes must cross the barrier of a thin layer of endothelial cells that separates hematopoietic compartments from the venous sinuses. The monolayer of endothelial cells has slits with a diameter ranging from 1 to 5 μm, which are smaller than the size of RBCs and passage through them verify the capacity of new cells for deformation. Approximately·200·10^9^ of RBC pass through these barriers each day (Vives Corrons et al., 2021).

It is noteworthy that genetically abnormal discocytes transform to spherocytes only after leaving the bone marrow hematopoietic compartments and entering the bloodstream. In the bone marrow of HS patients, the maturing erythrocytes show only minor shape changes. They evidently become more abnormal in shape shortly after release into circulation. The osmotic fragility of the newly generated erythrocytes is similar to the fragility of the majority of the cells in the peripheral blood. Newly appeared in the peripheral circulation red cells (labeled *in vivo* with Fe^59^) are characterized by some increase in fragility, but after approximately 10 days, they show a significant increase in osmotic and mechanical fragility (Griggs et al., 1960).

During a lifespan of about 120 days, RBCs are making in the bloodstream the way of about 300 km, repeatedly passing through arteries, arterioles, veins, venules and capillaries with diameters ranging from 3 to 8 µm. In healthy individuals, RBCs vary greatly in size with an average diameter of about 7.5 µm, maximum thickness of 2.0–2.5 µm, minimum thickness of 0.8–1 μm, volume between 83 and 98 μm^3^, and a surface of between 119 and 151 μm^2^. To pass through the capillaries, RBCs need the ability to deform reversibly. This is due to biconcave shape of these cells and the elasticity of the cytoskeleton that lines the plasma membrane. In RBCs, which vary markedly in size, surface area and volume are linearly related. The surface-to-volume ratio (S/V) is about of 1.56 meaning a ∼40% excess of surface area compared to a sphere of the same volume. This excess of surface area relative to volume allows RBCs to change shape without changing the volume when passing through capillaries. Over time, the size of red blood cells gradually decreases, the density increases, the deformability decreases, along with changes in other physicochemical and biochemical parameters, such as membrane transport and glucose metabolism. Aged RBCs exhibit significant biochemical changes that affect metabolic activity, leading to ATP depletion, increased oxidative modification of cytoskeleton and denaturation of hemoglobin (Vives Corrons et al., 2021; Garcia-Herreros et al., 2022).

The detection and subsequent removal of old, deformed and damaged red cells occurs in the spleen and liver. Erythrophagocytosis can also occur in the bone marrow, the place where RBCs have been generated (Vives Corrons et al., 2021). The passage of RBCs with genetic defects that weaken the cohesion of the plasma membrane to the cytoskeleton through the spleen can cause irreversible structural deformations in them: the loss of membrane fragments in the form of microvesicles (also called as extracellular vesicles or microparticles), and finally, transformation of discocytes into spherocytes. It can be concluded that the reduction in cell surface is the physical basis for spherocytosis (Lutz and Bogdanova, 2013; Li et al., 2018).

## 3 Spherocytosis

### 3.1 Clinical signs of the HS

HS is a group of diseases that vary greatly in clinical manifestations and severity, and is usually classified into 4 categories. 1) Mild or minor HS occurs in 20%–30% of patients and is characterized by compensated hemolytic anemia with hemoglobin levels close to normal (110–150 g/L), insignificant presence of spherocytes in blood smears, moderate splenomegaly, a small increase in the number of reticulocytes (3%–6%) and total bilirubin (17–34 μM). The normal values for reticulocyte and bilirubin levels are below 1.5% and 11 μM, respectively. Probably that the incidence of moderate spherocytosis is underestimated (Rocha et al., 2011). The presence of mild HS may be revealed with a delay, only after the appearance of gallstones or anemia induced by a viral infection such as parvovirus B19. 2) Moderate HS is the most common form of the disease (60%–70% of cases), characterized by a decrease in hemoglobin levels to 80–110 g/L, an increase in reticulocytes to 6%–10%, total bilirubin >34 μM, soluble transferrin receptor (sTfR) > 82.2 nM. Besides, the splenomegaly is present in approximately 50% of patients. 3) Moderate severe HS accounts for about 10% of all cases. The hemoglobin level is 60–80 g/L, reticulocyte content is more than 10%, bilirubin level is up to 51 μM. For anemia prevention, patients require regular blood transfusions. (iv) Severe HS accounts for about 3%–5% of patients. Hemoglobin levels are below 60 g/L, reticulocyte counts >10%, bilirubin concentration >51 μM, sTfR concentration >99.1 nM. To maintain hemoglobin levels above 60 g/L, patients also require regular blood transfusions. Most often, the inheritance of acute HS is not dominant. The disease is detected already in newborns (Da Costa et al., 2013; Perrotta et al., 2008). In most severe cases, in order to prevent anemia and chronic hemolysis, in addition to blood transfusion and, as a last resort, a spleen removal may be necessary. This surgery reduces the mechanical damage to spherocytic red cells, which are prone to generate microvesicles and thereafter have decreased elasticity and ability to deform but still functional in other ways (Shah and Vega, 2004; van Vuren et al., 2019).

### 3.2 Laboratory diagnostics of HS

A majority of patients with the dominant form of HS have family members who also suffer from this disease, and their examination facilitates diagnosis. The main symptoms of HS include anemia, jaundice, and gallstones. Laboratory blood tests usually reveal the presence of abnormal erythrocytes and microspherocytes, which are result of RBC fragmentation. There is also an increase in levels of total bilirubin, erythropoietin and reticulocytes, as well as lactate dehydrogenase activity. More detailed analysis of status of RBCs reveals a typical decrease in the elasticity of the plasma membrane as measured by osmotic fragility test (OFT). Other parameters that may be affected include increased mean corpuscular hemoglobin concentration (MCHC) and RBC size distribution width (RDW). It is worth noting that spherocytic red cells may also be present in other diseases, such as autoimmune hemolytic anemias, glucose-6-phosphate dehydrogenase deficiency and microangiopathic hemolytic anemias showing schistocytes and irregularly contracted cells that can mimic spherocytes, as well as thermal injuries, and venom poisoning (a secondary spherocytosis) (Perrotta et al., 2008; Bain, 2008; Lesesve et al., 2002). In addition, spherocytes are formed during long-term blood storage, when the morphology of RBCs irreversibly changes, the cells lose their membrane through vesiculation and a significant portion of the population becomes spherocytic (Flatt et al., 2014).

The diagnosis of HS can be complicated by several other hematological disorders. If HS occurs simultaneously with megaloblastic anemia caused by a deficiency of folic acid or vitamin B12, blood smears will show discocytes and macrocytes with normal center pallor, but few spherocytes. OFT curves are virtually normal. Deficiencies of folate or vitamin B12 are known to cause a delay in RBC maturation and the formation of relatively larger membrane area compared to normal. This then improves the S/V ratio and the osmotic resistance of these cells increases. Treatment with folic acid and vitamin B12 reverses all RBC parameters to the classic picture of HS (Blecher, 1988). Similarly, patients with both HS and β-thalassemia have milder clinical symptoms, and the degree of expression of one disease is counteracted by the other (Pautard et al., 1988; Habibzadeh et al., 2024). On the contrary, patients with both HS and sickle cell disease (SCD) have increased clinical severity. The combined effects of these two conditions result in decreased RBC deformability and subsequent splenic sequestration crisis. OFT may not be effective in these patients since the parallel effects of loss of cell surface area due to HS and the decrease in cell volume due to SCD lead to cells with normal S/V ratio and normal osmotic fragility. Coinheritance of SCD and HS is very rare (Warkentin et al., 1990; Selcuk Duru et al., 2008).

The basal principle behind the traditional laboratory tests of HS and other hereditary red cell membrane disorders exploit the reduced elasticity and integrity of the plasma membrane. These tests measure the rate of RBC lysis in different incubation conditions. The standard OFT measures the degree of hemolysis caused by hypotonic NaCl solutions at room temperature. The test determines the concentration of NaCl that causes 50% lysis of RBCs. The sensitivity of OFT can be improved by incubation of blood at 37°C for 24 h before testing (incubated OFT). All modifications of the OFT are based on the fact that RBCs of patients with HS undergo hemolysis at a higher concentration of NaCl than healthy cells. The incubated OFT is probably the most sensitive method for detection of silent carriers. The cryohemolysis test is based on the increased susceptibility of RBCs of HS patents to rapid cooling from 37°C to 0°C and following 10 min incubation in hypertonic conditions (0.7 M buffered sucrose solution). The results of cryohemolysis test represent the percentage of the lysed RBCs during a given time interval (Emilse et al., 2018; Shahal-Zimra et al., 2022; Wu et al., 2021; King and Zanella, 2013).

However, OFT shows false negative results in 10%–20% of HS cases, and normal indications do not exclude the diagnosis of HS. OFT can also be false negative in cases of iron deficiency and cholestatic jaundice. Dehydration of cells occurring in spherocytes can lead to normal OFT results for non-splenectomized HS patients. Furthermore, false positive results may be obtained for patients with hereditary elliptocytosis and autoimmune hemolytic anemia (Bolton-Maggs et al., 2004; Park, 2016).

The acidified glycerol lysis test (AGLT) is a reliable first-line method for detecting mild HS. This method is based on the affinity of glycerol for membrane lipids. Incubation of erythrocytes suspended in phosphate-buffered saline (pH 6.85) with 300 mM glycerol leads to the slow hemolysis of RBCs, measured by monitoring the decrease absorbance at 625 nm. The results are expressed as a time during which the 50% of erythrocytes undergo hemolysis. The diagnostic sensitivity of AGLT is better than that of OFT. Moreover, AGLT has a higher detection sensitivity for asymptomatic relatives of known affected individuals compared to the OFT (Bolton-Maggs et al., 2004). However, patients with autoimmune hemolytic anemias and chronic renal failure may also be positive for AGLT. On the contrary, when using OFT and AGLT, the diagnosis may be falsely negative due to the high number of reticulocytes in the blood, which unlike erythrocytes, do not have increased osmotic fragility (Wu et al., 2021; Bianchi et al., 2012; Ciepiela, 2018).

HS is also reliably detected by the reduced binding of eosin-5-maleimide (EMA) to the Lys430 residue of the transmembrane protein Band3, which is due to a decrease in its cellular level. EMA binding test can be attributed to the use of flow cytometry as a detection system, which analyzes single red cells in a sample. By contrast, other screening tests measure hemoglobin released from RBCs using spectrophotometry. The result can be masked by the ability of EMA to react to thiol groups of several other membrane proteins, such as rhesus factor (Rh), Rh-associated glycoprotein (RhAG), and CD47. Patients with hereditary elliptocytosis, Southeast Asian ovalocytosis, and hereditary pyropoikilocytosis may also be positive for EMA binding test. Anyway, the EMA binding test paired with AGLT can detect the majority of cases of HS. At the same time, when used separately, EMA and AGLT recognize 93% and 95% HS carriers, respectively (Wu et al., 2021; Bianchi et al., 2012). In comparison, the variants of osmotic fragility method have a sensitivity of 68% for fresh blood samples (OFT) and 81% for the incubated OFT approach. These methods are less effective in identifying compensated and almost asymptomatic cases of HS (53% and 64%, OFT and incubated OFT, respectively) (King and Zanella, 2013).

The method of osmotic gradient ektacytometry reliably distinguishes HS from other hereditary congenital hematological diseases, such as elliptocytosis, poikilocytosis, stomatocytosis and congenital dyserythropoietic anemia type II (CDAII). The Ektacytometry is based on measuring the osmotic fragility of low hematocrit RBCs suspension in a viscous medium under shear stress. Using a laser diffraction technique, ektacytometry measures the ability of erythrocytes to deform under changing osmotic conditions. This involves applying shear stress to a thin layer of cell suspension between two rotating surfaces, which leads to transformation of RBCs from the discoid-shaped cells into elliptical ones. The diffraction pattern reflects the shape and geometry of deformed cells and is usually expressed as a dimensionless elongation index (EI). This method is only available for specialized laboratories and is not commonly used as a routine diagnostic tool. Relatively more available is a modified ektacytometer, the laser-assisted optical rotational cell analyzer (LORCA) (Huisjes et al., 2018; Baskurt et al., 2009; Zaninoni et al., 2018).

Another approach is based on microfiltration of blood or its passage through channels with different pore sizes, which can distinguish spherocytes from discocytes and characterize the deformability of the RBC population. While normal RBCs easily pass through filters with pore diameters of 3–3.5 μm, the cells from patients with HS are trapped when passing through these filters, or even when they pass through filters with a larger pore diameter of 4–5 μm (Jandl et al., 1961; Oonishi et al., 1997; Lisovskaya et al., 1999; Prudinnik et al., 2022; Khanna et al., 2002). Various microfluidic devices simulate the splenic microcirculation. For example, a suspension of RBCs is injected into a network of microchannels, where the RBCs flow through from circular capillary tubes or slits with a width ranging from 2- to 5 μm. This technique also allows us to study the mechanism of the retention of spherocytes and damaged RBCs in the spleen (Huisjes et al., 2018; Picot et al., 2015; Faivre et al., 2020; Ugurel et al., 2022; Garcia-Herreros et al., 2022).

Relatives of patients with a recessive form of heredity are most often asymptomatic carriers. About 1% of the population are estimated to be silent carriers of HS (Perrotta et al., 2008; Godal and Heistø, 1981; Eber et al., 1992). The definitive confirmation of HS can be achieved by identifying genetic mutations that cause this disease through sequencing of HS associated genes. This can also be done through the detection of structural defects and changes in the expression of corresponding proteins using the of denaturing electrophoresis technique (SDS-PAGE). Protein extracts from erythrocyte shadows are separated electrophoretically in a gradient polyacrylamide gel. The range of alterations in the expression of the HS-associated proteins will be discussed below. The results of the SDS-PAGE analysis may be misinterpreted if there are high levels of reticulocytes in the blood. Moreover, about 10% of patients do not have a detectable deficiency in HS-associated proteins. In general, the use of laborious methods such as ektacytometry, SDS-PAGE, and genetical analysis is only required in certain atypical cases of HS (Perrotta et al., 2008; Silva et al., 2022).

## 4 Proteins of the erythrocyte membranes and cytoskeleton

**FIGURE 1:**
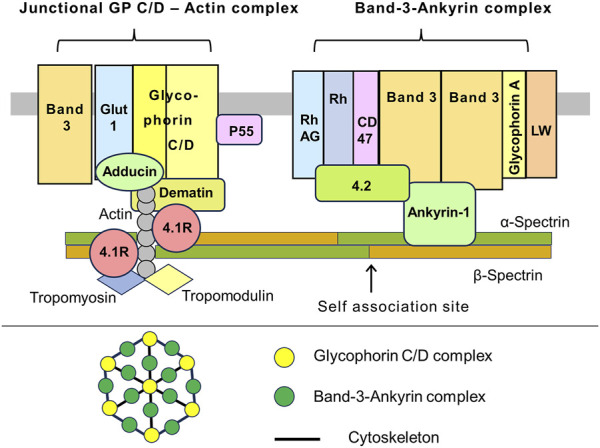
A schematic representation of the interaction between the RBC membrane and the underlying cytoskeleton. Cross-section of the membrane. The Band 3 – Ankyrin-1 complex is centered by a Band 3 tetramer. Among the Band 3 associated proteins, there is ankyrin-1, which also binds to the β-spectrin chain and protein 4.2. Glycophorin A occurs as a dimer. Apparently that the Band 3 complex and transmembrane Rh complex form a higher order macromolecular complex. The Rh complex consists of the rhesus factor proteins, RhAG, CD47 (also known as a thrombospondin receptor), and glycoprotein LW, also known as intercellular adhesion molecule-4 (ICAM-4). CD47 and Rh interact with ankyrin-1 via protein 4.2. The junctional complex. Pivotal protein 4.1R interacts with N-terminal domain of β-spectrin. This region contains short filaments of the F-actin and actin-binding proteins like dematin, tropomyosin, and tropomodulin. The transmembrane part of the junctional complex includes glycophorins C/D (GP C/D), glucose transporter 1 (Glut1), and protein p55. In addition, the complex interacts with a Band 3 population (presumably dimers), which is separate from the Band 3 – Ankyrin complex. The α_2_β_2_ tetramers of spectrin form a dense network lining the inner surface of the lipid bilayer. Spectrin tetramer is shown in a straight configuration, although they may be curved in reality (upper drawing). Top view of the membrane. The membrane skeleton is a network formed by quasi hexagonal units centered around actin filaments that are attached to glycophorins C/D. Band 3 and ankyrin interact with C-terminal part of β-spectrin, approximately in the middle of the α_2_β_2_ tetramers. The junctional complex is located at the nodes of hexagons. The exact relative positions of the depicted proteins to each other within the complexes may be unknown. The proteins and lipids are not drawn in their actual scale and shape (lower drawing). This illustration is a merged figure adapted from both (Delaunay, 2007; Perrotta et al., 2008; Lux, 2016).

In RBCs about 23% of the plasma membrane area is covered by the external domains of transmembrane proteins, whereas about 50% of the plasma membranes of most animal cells are occupied by proteins (Dupuy and Engelman, 2008). The attachment of the spectrin network to the membrane is mediated by two multicomponent modules: 1) Band 3 - ankyrin complex and 2) glycophorin C/D–actin or junctional complex). Erythrocyte Band 3, also known as SLC4A1 and AE1, is the main RBC membrane protein with approximately 1.2·10^6^ copies per cell. Band 3 consists of two functionally distinct parts. 1) An N-terminal cytoplasmic domain with attachment site for spectrins, ankyrin, protein 4.2, protein 4.1R, glycolytic enzymes, and deoxyhemoglobin. 2) The C-terminal transmembrane domain with a function of hydrocarbonate/chloride anion exchanger. The Band 3 complex is associated with the transmembrane Rh complex which includes Rh and RhAG proteins which acts as ammonium transporter. Also, Band 3 is associated with CD47 (thrombospondin receptor), glycophorin A, and Landsteiner-Wiener (LW) glycoprotein (Delaunay, 2007; Perrotta et al., 2008; Lux, 2016; Tse and Lux, 1999; Narla and Mohandas, 2017) (Figure 1).

The composition and distribution of lipids in RBC membrane are quite complex. The inner leaflet of plasma membrane contains phosphatidyl serine. The outer leaflet contains two types of submicrometric domains that are differently enriched with either cholesterol or sphingomyelin. Cholesterol- and sphingomyelin-enriched domains are present in unequal amounts in distinct areas of the erythrocyte biconcave membrane. Upon membrane deformation as RBCs pass through capillaries, cholesterol-enriched domains accumulate in areas with high curvature. During restoration of shape after deformation, the abundance of sphingomyelin-rich domains is increased in the concavity of the RBCs. This process is dependent on Ca^2+^. It is likely that, along with the aging of RBCs, both lipid domains become sites of membrane loss by vesiculation (Leonard et al., 2017; Bennett and Healy, 2008). Vesiculation allows RBCs to shed membrane fragments that contain markers that promote phagocytosis, such as phosphatidylserine and denatured Band 3 protein. This process is thought to protect from premature elimination RBCs during their lifespan (Garcia-Herreros et al., 2022; Willekens et al., 2008).

### 4.1 α- β-Spectrins

In erythrocytes, the base of cytoskeleton are the dimers of α- and β-spectrin which are arranged in a tandem manner. That is, the N-end of the α-spectrin chain interacts with the C-end of the β-spectrin chain within the same dimer. Each chain contains multiple repeats, with a length of approximately 5 nm and consisting of around 106 amino acids. α-Spectrin and β-spectrin contain 21 and 17 numbered repeats, correspondingly. Pairs of dimers are joined side by side in an antiparallel manner to form α_2_β_2_ tetramers, which in turn, in higher order structures, form the sides of equilateral triangles. These triangles are further connected into quasi-hexagonal structures. *In vivo* the average length of a spectrin tetramer, which corresponds to the half of distance between actin filaments in two neighboring junctional complexes, is approximately 32 nm, and the area of the quasi-hexagon is about 3,500 nm^2^. Remarkably that the end-to-end length of the fully extended tetramer should be about three times more than 32 nm. This discrepancy suggests that, in natural conditions, spectrin tetramers coil around each other. Spectrin oligomers higher than tetramers (hexamers and octamers) are also present on the membrane, although almost 95% of the spectrins exist in the form of tetramers. A continuous network of hexagons completely covers the inner surface of the plasma membrane of erythrocytes that lack an intracellular cytoskeleton (Lux, 2016; Machnicka et al., 2014; Johnson et al., 2007).

**FIGURE 2:**
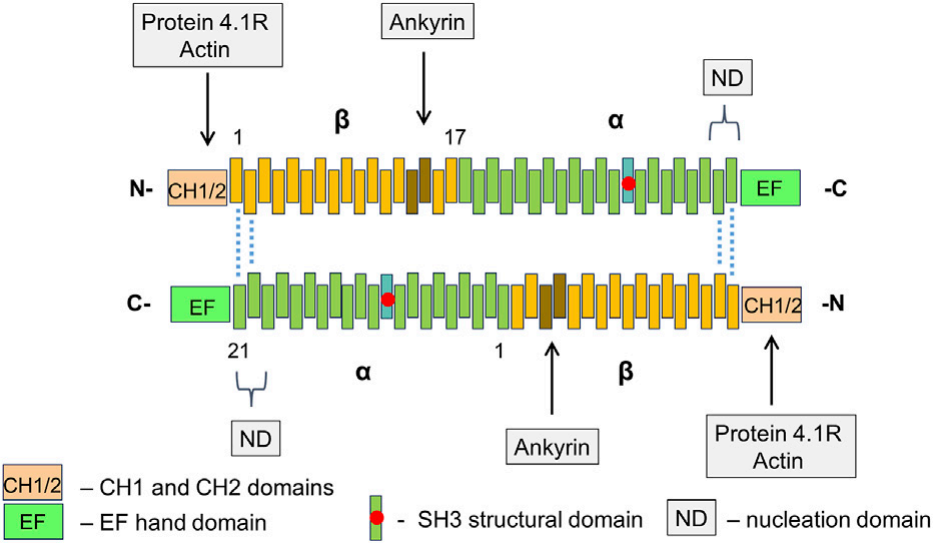
The structure of RBC spectrin tetramer The α_2_β_2_ tetramers of spectrin form a network lining the inner surface of the lipid bilayer. In spectrin dimers, the α- and β-chains are arranged tandemly. Two dimers connect side-by-side in an antiparallel manner with the N-terminals region of α-chains facing C-terminal regions of β-chains. Each chain contains a series of repetitive domains, each consisting of 106-amino acids, as well as additional specialized functional domains. α-Spectrin has 21 numbered repeats (α1 – α21), plus a partial repeat (α0) at the N-terminus. The repeat α10 comprises the src homology 3 (SH3) subdomain. β-Spectrin has 16 numbered repeats (β1 – β16) and a partial C-terminal repeat (β17). Dimer-tetramer self-association of α- and β-spectrin chains takes place at a nucleation domain (ND) near the edges of spectrin tetramers (repeats β1 and β2 pair with α21 and α20). Actin and protein 4.1R bind to calponin homology (CH) actin-binding domains at the N-end of β-spectrin. Adjacent EF hand domain on α-spectrin binds Ca^2+^ ions.

**FIGURE 3:**
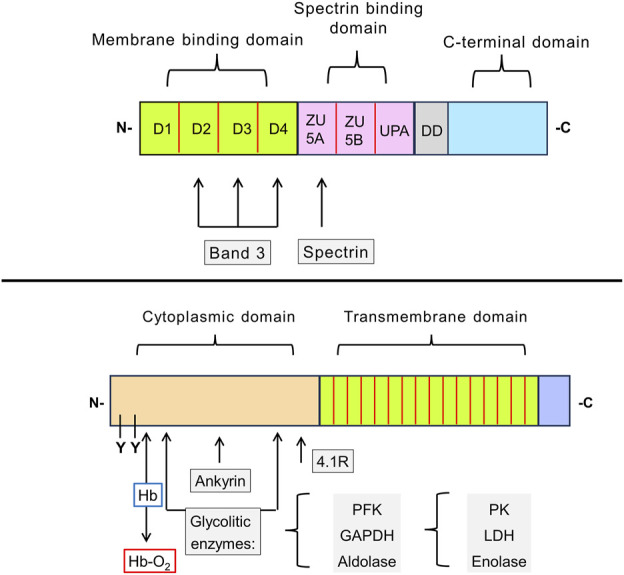
Schematic representation of the structure of erythroid ankyrin and Band 3 proteins. Ankyrin-1 consists of membrane binding, spectrin binding, and C-terminal domains. The N-terminal membrane binding domain has 4 subdomains (D1-D4). There are two binding sites for the Band 3 protein, one located in the subdomain D2, D3, and D4. The spectrin binding domain contains three subdomains, of which ZU5A contains the binding site for β-spectrin (repeats β14 and β15). The function of presumably regulatory C-terminal domain and neighboring so-called death domain (DD) remains unclear (upper drawing). Band 3 contains two functionally distinct domains: 1) responsible for binding of cytoplasmic proteins domain and 2) a transmembrane domain that forms the anion-exchange channel. In the cytoplasmic domain, the glycolytic enzymes (PFK, aldolase, and GAPDH) bind to amino acid sites located nearby in the folded protein. PK, LDH, and enolase do not interact directly with Band 3, but they form a complex with the three glycolytic enzymes mentioned. These enzymes are displaced by deoxyhemoglobin, which also binds to the N-terminus of the Band 3, or by phosphorylation of tyrosine residues (Y8 and Y21). Ankyrin and protein 4.1R also interact with the cytoplasmic domain of Band 3. The transmembrane domain is composed of 16 subdomains that form ion channels responsible the intake of bicarbonate ions from the blood (lower drawing). Abbreviations in the figure: GAPDH, glyceraldehyde-3-phosphate dehydrogenase; Hb, hemoglobin; Hb-O_2_, oxyhemoglobin; LDH, lactate dehydrogenase; PFK, phosphofructokinase; PK, pyruvate kinase; Y, tyrosine phosphorylation site.

Spectrins fulfill many functions in the physiology of RBCs. They are involved both in the formation and maintenance of the membrane structure, as well as in interaction with membrane ion channels, adhesion molecules, receptors, and transporters. α- and β-spectrins each contain various functional domains. F-actin and protein 4.1R bind to CH domains at the N-terminal end of β-spectrin at the vertices of hexagons. Adducin also binds in the same region. Protein 4.2 and Ca^2+^ ions bind to a neighboring domain on α-spectrin. Ankyrin-1 binds to β-spectrin repeats β14-β15. Interactions within the spectrin α1β1 dimers outside the nodes are relatively weak allowing two chains to slide past each other when the spectrin molecules bend and extend during membrane deformation. Moreover, tetramers can dissociate and then recover under physiological conditions. In addition, some of the helical segments of the repeats forming spectrin chains are probably unstable at physiological temperatures and, when stressed, may melt, partially unfold and extend during RBC deformation, contributing to their flexibility. This model suggests that in RBCs, the activity of glycolytic pathway is stimulated under hypoxic conditions. These conditions occur, for example, when abnormal RBCs are trapped in the red pulp of the spleen (Figures 1–3) (Lux, 2016; Bennett and Healy, 2008; Machnicka et al., 2014; Johnson et al., 2007).

### 4.2 Band 3 protein

About 40% of the Band 3 molecules are tetramers forming the complexes with ankyrin and other integral proteins located near the spectrin self-association site. An approximately similar fraction of the Band 3 molecules, probably dimers, is located near the spectrin-actin junction and binds to spectrin via protein 4.1R and adducin. These two complexes, along with their associated proteins, may be large enough to sometimes contact each other. The remaining Band 3 dimers float freely in the lipid bilayer (Kodippili et al., 2012).

The protein complexes, which include Band 3, have a flexible composition. Some proteins are present in much smaller quantities than others. The amount of p55, adducin, and dematin is insufficient to interact with all the spectrin/protein 4.1R-actin complexes, which can lead to variations in the composition and lateral mobility of the Band 3-ankyrin and glycophorin-C-actin complexes (Lux, 2016; Gokhin and Fowler, 2016).

It was shown that three glycolytic enzymes, phosphofructokinase, aldolase, and glyceraldehyde-3-phosphate dehydrogenase, bind to the N-terminus of Band 3. Several other glycolytic enzymes bind to the Band 3 indirectly as part of the complex. The enzymes are inactive upon binding but are displaced and activated either by deoxyhemoglobin, which also binds to the N-terminal domain, or by phosphorylation of two tyrosine residues within this domain. Band 3 phosphorylation is mediated by the src family non-receptor tyrosine kinase Syk (Figures 1, 3) (Rifkind and Nagababu, 2013; Chu et al., 2008; Nunomura and Takakuwa, 2006; Noomuna et al., 2020; Campanella et al., 2005; Puchulu-Campanella et al., 2013).

As with the glycolytic enzymes, ankyrin is also displaced from Band 3 by deoxyhemoglobin. Deoxyhemoglobin has a weak affinity to Band 3, but this is compensated by the high concentration of hemoglobin in RBCs. Approximately half of Band 3 molecules become bound to deoxyhemoglobin instead of ankyrin when the cells are deoxygenated. This weakens the membrane–cytoskeletal cohesion, and during brief deoxygenation it may improve blood flow in hypoxic areas. However, prolonged deoxygenation, such as when RBCs are trapped in the spleen, can induce membrane vesiculation. RBCs captured in the spleen parenchyma (see below) are evidently located in a deleterious environment, which eventually leads to alterations in the cells. This process is known as “conditioning” (Griggs et al., 1960; Lux, 2016; Stefanovic et al., 2013).

Tyrosine phosphorylation of Band 3 reduces its affinity for ankyrin, inducing the cleavage of the Band 3-ankyrin bridge, which leads to significant changes in the membrane state, an increase in the lateral mobility of Band 3 in the lipid bilayer, vesiculation of the plasma membrane, and a decrease in its surface area. It has been shown that in patients with SCD, autoimmune hemolytic anemias, and malaria, the constant accumulation of oxidized and denatured hemoglobin induces phosphorylation of Band 3 which leads to destabilization of the membrane. Band 3 phosphorylation and subsequent abnormalities can be suppressed by pharmacological inhibition of Syk (Noomuna et al., 2020; Pantaleo et al., 2016; Puchulu-Campanella et al., 2016). The involvement of Syk in HS has not yet been studied. Nevertheless, HS is associated with the appearance of hyperchromic RBCs due to hemoglobin denaturation, which is a process associated with Syk activation (Rooney et al., 2015; Berrevoets et al., 2021).

### 4.3 Glycophorin C/D and associated proteins

Glycophorin C/D is an integral membrane protein that is a major component of the junctional complex, which connects plasma membrane to the cytoskeleton. The junctional complex consists of several components, including F-actin, which is composed of γ-actin monomers polymerized into short filaments. The actin filaments lie parallel to the membrane. Other components of the junctional complex include adducin, dematin (also referred as Band 4.9), protein 4.1R, tropomyosin, and tropomodulin. Adducin is a multifunctional protein containing an α subunit and either a β or γ subunit. Dematin, a multifunctional protein with two binding sites for actin, also binds to spectrin, enhancing spectrin-actin interaction. This function is lost when dematin is phosphorylated by cAMP-dependent protein kinase (PKA). Protein 4.1R is encoded by the *EPB41* gene. The protein contains two main functional domains. (i) The N-terminal membrane-binding domain, which contains binding sites for p55 and calmodulin (CaM), as well as to the cytoplasmic segments of integral membrane proteins, such as Band 3, glycophorin C/D, and CD44. (ii) The internal domain which interacts with spectrin and actin that is critically important for membrane stability (Figure 2) (Delaunay, 2007; Lux, 2016; Cilek et al., 2024). Hereditary defects in 4.1R lead to abnormal shape of RBCs and decreased mechanical stability of membranes (Nunomura and Takakuwa, 2006). It is interesting to note that glycophorin C/D does not seem to be crucial for maintaining the RBC morphology. Patients with complete absence of glycophorin C have, at most, a mild hereditary elliptocytosis (Delaunay, 1995).

## 5 The genetic basis of HS

The genetic mutations that cause HS are extremely heterogeneous. These mutations lead to membrane instability, shrinkage of membrane due to the release of hemoglobin-free vesicles, followed by a decreased surface area-to-volume ratio, an increase in cell density and, finally, the transformation of normal biconcave discocytes into spherocytes. According to atomic force microscopy data, spherocytes do not have a spherical shape, but rather resemble an oblate spheroid (Delaunay, 2007; Perrotta et al., 2008; Starodubtseva et al., 2019).

In most cases, HS is caused by mutations in at least one of the five genes encoding α- and β-spectrins, ankyrin-1, Band 3 and 4.2 proteins. About 25% of HS cases are associated with more than one mutation of these genes. Defects in the proteins encoded by these genes lead to a decrease in the attachment of the plasma membrane to the cytoskeleton, which occurs for two reasons: 1) weakening of vertical bonds due to mutations in the Band 3 protein and 2) a decrease in the density of the cytoskeleton underlying the membrane due to mutations in spectrins, ankyrin or protein 4.2 (Bianchi et al., 2012; Vercellati et al., 2022).

In addition to abnormalities of the cytoskeleton and membrane proteins, HS is associated with disturbed ion balance, an increased level of Ca^2+^, and a reduced concentration of K^+^. The latter may be related to activation of KCNN4 (or Gardos) channels (Huisjes et al., 2018). In HS, erythrocytes are characterized by the leakage of K^+^ ions, which is not compensated by an parallel increase in the activity of Na^+^,K^+^-ATPase (by about 40%). Spherocytes have total concentration of monovalent cations, including K^+^, that is about 13 mM lower than in normal cells, which leads to the cell dehydration and increased density (Vives Corrons and Besson, 2001).

### 5.1 Mutations of the *SPTA1* gene (α-spectrin)

Mutations are observed in about 5% of all patients with HS. The inheritance of HS mediated by mutations of *SPTA1* is recessive. Since the production of α-spectrin during erythropoiesis happens in about 4-fold excess compared to its content in mature RBCs, a mutation in one of the alleles usually has no consequences. Accordingly, the disease manifests itself in the cases of compound heterozygotes or hemizygotes. In patients with HS, the level of α-spectrin, as determined by SDS-PAGE, is 50%–75% that of healthy controls. The most common abnormal allele α^LEPRA^ contains the intron mutation (a c → t transition at position 99 of intron 30 results in the aberrant splicing, frameshift and premature termination of translation). Nevertheless, 16% of the primary transcripts escape the abnormal splicing. Therefore, the allele α^LEPRA^ can compensate for a weak or even null allele on the homologous chromosome. Homozygous mutations of the *SPTA1* gene are lethal. Along with HS, *SPTA1* mutations can cause hereditary elliptocytosis and hereditary pyropoikilocytosis (Delaunay, 2007; Maillet et al., 1996; Wichterle et al., 1996).

### 5.2 Mutations of the *SPTB* gene (β-spectrin)

Mutations are observed in about 25% of all patients with HS. The inheritance of HS, mediated by *SPTB* gene, has a dominant pattern. SDS-PAGE analysis reveals a decrease in the cellular content of β-spectrin by 15%–40% compared to healthy control. In the presence of the *SPTB* mutations, the cytoskeleton becomes less dense. Production of β-spectrin in erythroblasts exceeds its content in RBCs which can compensate the effect of weak mutations. Homozygous mutations of *SPTB* gene are unknown. In addition to HS, *SPTB* mutations can also cause hereditary elliptocytosis and hereditary pyropoikilocytosis (Delaunay, 2007; He et al., 2018).

### 5.3 Mutations of the *ANK1* gene (ankyrin-1 or rAE1)

Mutations of *ANK1* gene are the most common among all cases of HS and are observed in about 40% of patients. The inheritance of HS mediated by mutations of *ANK1* gene is dominant. All the main types of mutations, including frameshift, nonsense, abnormal splicing, missense, as well as mutations in the promoter region, have been identified. SDS-PAGE analysis detects a decrease in the levels of ankyrin in RBCs (15%–50% below the normal), as well as decrease in spectrins and protein 4.2 as a secondary effect. Homozygous mutations of the *ANK1* gene are unknown. Homologous genes, *ANK2* and *ANK3*, have also been identified. In addition to RBCs, the *ANK1* gene is expressed in the brain and the kAE1 protein, a truncated isoform of the ankyrin-1/rAE1, is expressed in kidneys (Da Costa et al., 2013; Delaunay, 2007; He et al., 2018; Sharma et al., 2020).

### 5.4 Mutations of the *SLC4A1* gene (Band 3)

Along with *ANK1*, the *SLC4A1* mutations are the most common cause of HS, accounting for approximately 25% of all cases. The inheritance of HS mediated by *SLC4A1* gene mutation is dominant. Mutations of various types have been identified in *SLC4A1* gene, and they located in all domains of the Band 3 protein. SDS-PAGE analysis reveals a decrease in Band 3 levels (35% below the normal) and a secondary decrease in the level of protein 4.2. Rare homozygous mutations of the *SLC4A1* gene have been revealed, including the Coimbra mutation (Val488Met), which causes an extremely severe form of HS, associated with impaired renal anion metabolism and kidney damage (distal renal tubular acidosis, DRTA) (Delaunay, 2007; Eber and Lux, 2004; Wang et al., 2021).

### 5.5 Mutations of the *EPB42* gene (protein 4.2) and Rh genes

The inheritance of HS, mediated by the *EPB42* gene mutations, is recessive. These mutations account for mild to moderate cases of HS. Mutations of *EPB42* are most common in Japan, accounting for 45%–50% of all cases of HS in this country (Delaunay, 2007). Less than 1% of HS cases are caused by Rh deficiency. The absence of Rh expression or significantly reduced RhAG expression are associated with mild-to-moderate spherocytic hemolytic anemia. About 10% of cases have an undefined molecular basis for the disease (Table 1) (Narla and Mohandas, 2017).

**TABLE 1:** Characterization of molecular defects in HS.

Molecular defect	Occurrence in HS population	Heredity	Prevalent mutations	Protein expression	Disease severity	Cytology
Ankyrin-1	40%–65% in Europe and United States of America; 5%–10% in Japan	AD, AR, *de novo*	AD: null mutations, AR: missense or promoter	Spectrin, ankyrin-1, 15%–50%	Mild to moderately severe	Spherocytes
Band 3	20%–35%	AD	Null mutations	Band 3, 15%–35%	Mild to moderate	Spherocytes, rare mushroom-shaped cells
α-Spectrin	<5%	AR	α^LEPRA^ allele and null mutations	α-Spectrin, 50%–75%	Severe	Spherocytes, poikilocytes
β-Spectrin	15%–30%	AD, *de novo*	Null mutations	β-Spectrin, 15%–40%	Mild to moderate	Spherocytes, acanthocytes, echinocytes
Protein 4.2	<5% in Europe and United States of America; 45%–50% in Japan	AR	Missense	Protein 4.2, 95%–100%	Mild to moderate	Spherocytes, ovalo-stomatocytes

Abbreviations in the Table: AD, autosomal dominant; AR, autosomal recessive (Da Costa et al., 2013; Eber and Lux, 2004; Perrotta et al., 2008).

## 6 Hereditary spherocytosis and the spleen

The spleen is a lymphoid organ divided into two functionally separate compartments, the white pulp and the red pulp. The white pulp is responsible for initiation of immune reactions, while the red pulp filters the blood and removes old or damaged erythrocytes, encapsulated bacteria, blood-borne pathogens, and cellular debris. Two distinct mechanisms lead to the filtering of erythrocytes in the red pulp: 1 physicochemical filtration, which involves the adherence of surface-altered RBCs to macrophages followed by removal of adhered RBCs through phagocytosis, and 2) mechanical filtration, where the slits of the sinus wall act as a barrier that prevents RBCs with abnormal size, shape, and deformability from returning to general circulation (Li et al., 2018; Mebius and Kraal, 2005). The blood flow through the spleen is approximately 300 mL/min, which is approximately equal to 6% of the total cardiac flow. During the day, each RBC passes through the spleen about 50–80 times (Dao et al., 2021; Kashimura, 2020).

Part of the arterioles of the spleen pass into the venous sinuses forming a closed circulation, through which 80%–90% of the total blood flow in the spleen passes. Another part of the arterioles forms an open blood stream, transferring blood to the red pulp (parenchyma, or reticular meshwork), which is enriched with macrophages. The red pulp is divided into isolated compartments, known as cords, that surround the venous sinuses and account for approximately 75% of the volume of the spleen. In the red pulp area, blood flows slowly from the arterioles towards venous sinuses. During this passage, macrophages recognize and remove senescent or damaged erythrocytes through phagocytosis. Approximately 0.9% of the RBC population are destroyed every day by macrophages. Additionally, spleen macrophages can remove denatured hemoglobin aggregates (Heinz bodies) and other inclusions from erythrocytes, without destroying them. This includes such inclusions as Howell-Jolly and Pappenheimer bodies (Mebius and Kraal, 2005).

**FIGURE 4:**
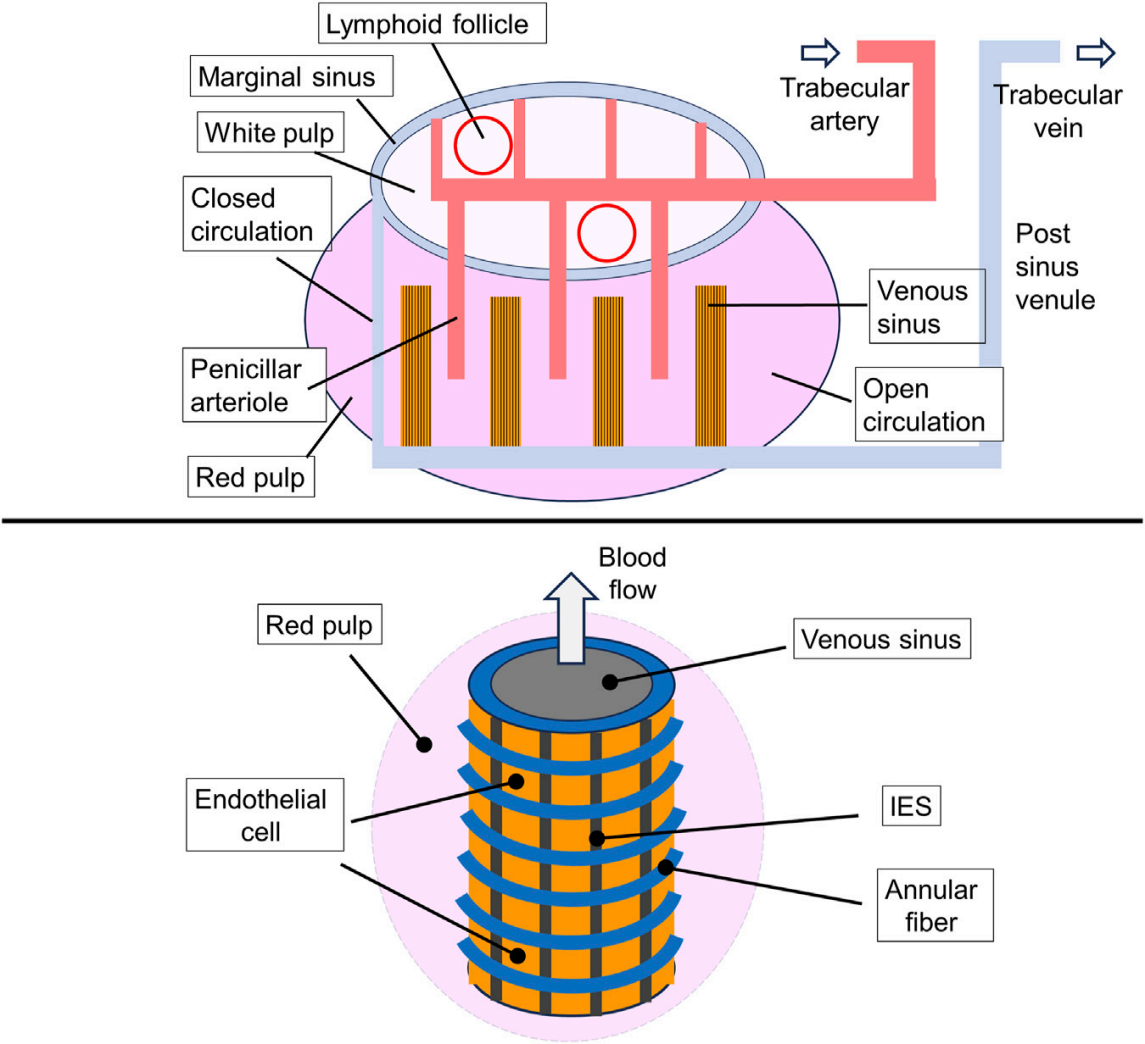
General representation of the microcirculation within the spleen. A map of the main spleen compartments. Afferent splenic (or trabecular) arteries branch into central arterioles that enter the white-pulp, which consists of an area containing T cells and dendritic cells (DCs) as well as lymphoid follicles that accumulate activated B cells. Arterioles end in white pulp capillaries, from where the blood enters the venous sinuses which gather in the efferent splenic vein, forming the closed blood circulation pathway. The open circulation consists of the flow of RBCs through penicillar arterioles opening into the reticular meshwork of the red pulp followed by filtration through the interendothelial slits (IES) in the venous sinuses. The interior blood pressure in red pulp is higher than that in the sinus lumen that determines the direction of RBC flow (upper drawing). A scheme of the venous sinus. The venous sinuses are formed by spindle shaped endothelial cells supported by ring (or annular) fibers. Stress fibers (not shown) extend under the basal plasma membrane of the endothelial cells, running parallel to the cell axis (lower drawing). This illustration is a merged figure adapted from both (Mebius and Kraal, 2005; Neri et al., 2021).

The endothelial cells lining the sinuses, that have an average diameter of approximately 13 μm, are arranged in parallel lines with a thickness of one layer of cells. These lines are separated by longitudinal slits and bonded by longitudinal (or stress) and annular (or ring) fibers, which are formed by components of the intercellular matrix. The stress fibers also contain nonmuscle myosin- and actin-like filaments. In some mammals, such as dogs, whales, and horses, the slits can contract and their width can be regulated (Figure 4). Apparently, the local contraction of a pair of these fibers causes the underlying endothelial cells to bend, leading to the opening of an internal venous slit, while next-neighbor slits may be narrowed. It seems likely that this pathway may become activated in the case of trapping of abnormal RBCs in the slits (Dao et al., 2021; Mebius and Kraal, 2005; Moreau et al., 2023; Pivkin et al., 2016; Neri et al., 2021; Drenckhahn and Wagner, 1986).

Interendothelial slits (IES) have the following parameters: height on average of 0.65 µm with a spread of 0.25–1.2 µm; endothelial thickness: on average of 1.89 µm with a range from 0.9 to 3.2 µm; slit width of 2–3 μm; annular fiber width of 1 µm (Li et al., 2018; Pivkin et al., 2016; Deplaine et al., 2011). In the rat spleen the mean number of IES per 100 μm^2^ is 8.0 with a range from 3.3 to 16.2. The pressure difference between the red pulp and the venous sinus is estimated at 130–200 Pa. In humans, the average time for a red blood cell to pass through IES is estimated to be 0.1–0.2 s (Dao et al., 2021). A microscopic examination of the blood flow through the spleen of an anesthetized rat revealed that only approximately 20% of the IES actually allowed the passage of RBCs during the observation period (∼5 min) (MacDonald et al., 1987). Worth to note that IES are significantly narrower and shorter than the capillaries of the blood circulatory system. The early *in vivo* experiments conducted on anesthetized rats showed a significant variation the time required to RBCs to transit the IES. This is reflected in the large difference between the median (0.23 s) and the mean (1.7 s) values. Apparently, these variations are at least partly due to differences in the size of the slits and heterogeneity of the erythrocyte population (Dao et al., 2021; Deplaine et al., 2011).

When passing through the slits, RBC with an average width and thickness of approximately 7.5 and 2 μm, respectively, must deform and at a certain moment, appears to take on the shape of a dumbbell (in other words, a form of two tether-connected spheres). To pass through IES, the surface of the RBCs must be 15% larger than the total surface area of two spheres of the same size. The surface area-to-volume ratio of 1.56 available in standard erythrocytes, meets this requirement. Significant deformation of the cells suggests that the local unfolding of spectrin is necessary for the passage of erythrocytes through the slits (Machnicka et al., 2014; Johnson et al., 2007; Moreau et al., 2023). It should be noted that, according to *in silico* experiments, RBCs were able to pass through the significantly narrower slits of IES-like constructions at 37°C, than at 25°C (the values of the minimal slit width were <0.3 and >0.5 μm, respectively). This suggests that the increase of RBC deformability at 37°C reflects the needs for the unfolding of the spectrin network or involvement of the another, currently unidentified, thermally activated mechanism. During the passage of RBCs through IES, a gradual increase in the concentration of free intracellular calcium was detected. This event is unrelated to the activation of Ca^2+^ channels. The RBCs are characterized by a significant exceed of total and mostly bound intracellular calcium over to free calcium. While the total Ca^2+^ concentration in RBCs reaches 5.7 μM, the basal free Ca^2+^ concentration in the cells of healthy donors is in the range of 30–60 nM (Moreau et al., 2023; Bogdanova et al., 2013).

**FIGURE 5:**
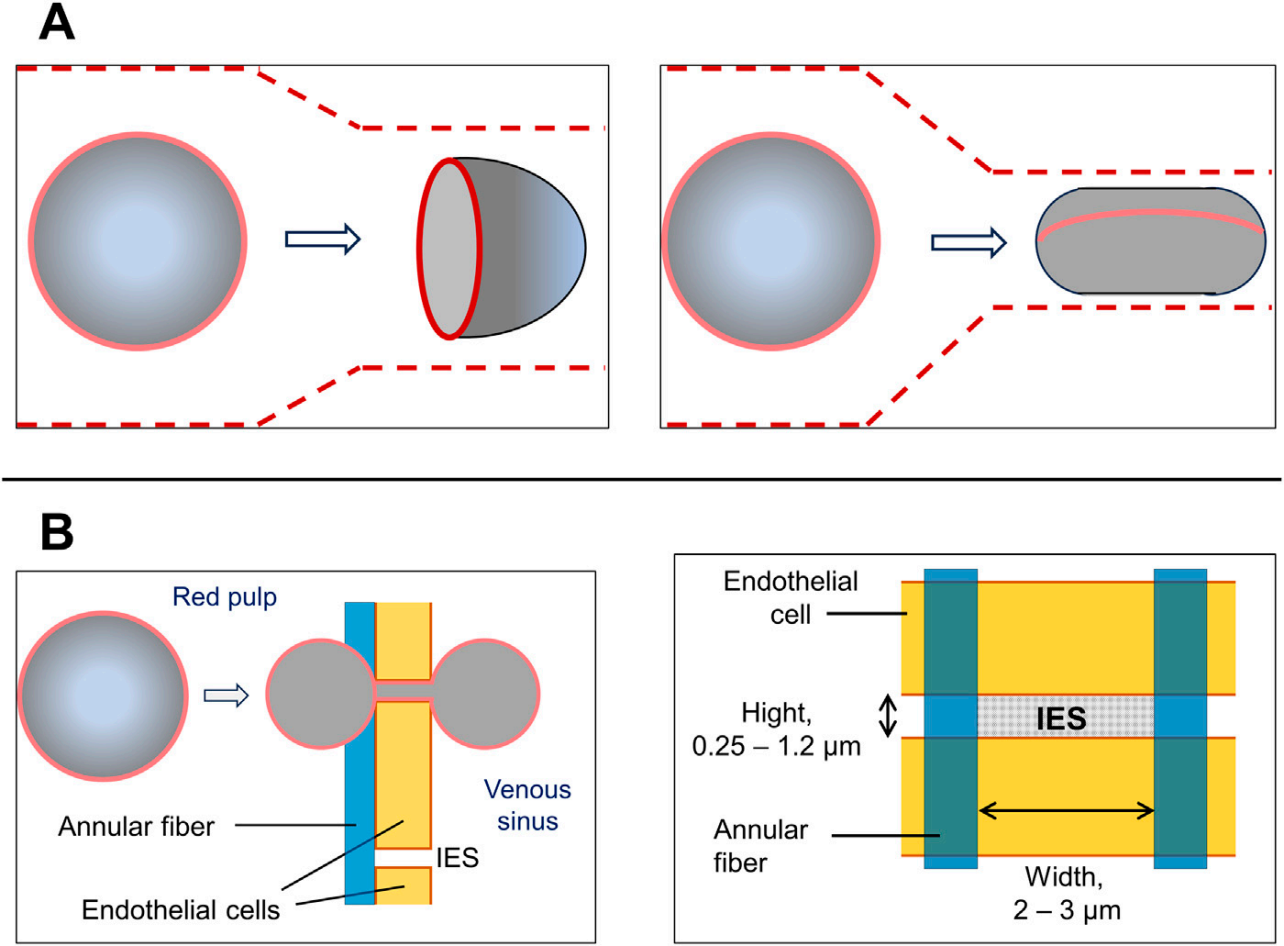
Comparison of RBC deformation during the passage through capillaries and spleen IES. **(A)** A scheme of an RBC transit through a capillary. Depending on the diameter of the capillaries, RBCs acquire either an axisymmetric “parachute-like” (left drawing) or cylindrical “cigar-like” shape (right drawing). The latter occurs when they pass through capillaries with almost minimal diameters (about 3 μm). RBC shapes in capillaries also depends from the stream velocity and shear forces. Capillaries are depicted by the dashed lines. **(B)** A scheme of an RBC transit through the interendothelial slit (IES) (left drawing). Apparently, in the middle of the passage, the red cells are divided into two equal spheres located on either side of the slit and joined by a thin connection. At this moment red cell is divided equally into two equal spheres on both sides of the slit. This configuration requires a high surface area-to-volume (S/V) ratio, which is inherent for discocytes, but not for spherocytes. In circulating healthy RBCs, the observed S/V ratio is approximately 1.56 which is not more than 15% larger than the minimum value that allows the formation of two equal spheres. Solid red lines indicate the edges of the biconcave disks of RBCs. The concave area of RBCs is depicted as less shaded. Blood flow is from left to right. Right drawing: a scheme of IES.

At the same time, the passage of RBCs through the more extended than IES artificial capillaries of microfluidic devices or capillaries *in vivo* results in a transient increase in the intracellular concentration of free Ca^2+^. This process appears to be mediated by the activation of mechanosensitive channels, such as Piezo1, which in turn activate the Gardos channel. Thereby mechanical stimulation leads to an increase in intracellular Ca^2+^. Interestingly, inhibitors of these channels (GsMTx-4 and TRAM-34) significantly reduce the ability of RBC to pass through capillaries (Danielczok et al., 2017). When RBCs pass through capillaries, they undergo significant morphological changes that differ from those that occur when they pass through the IES. In capillaries, RBCs become folded and take on an ellipsoidal (or cigar-shaped) form. Apparently, the minimum diameter of a capillary that an RBCs can pass through is approximately 3.6 μm (Figure 5) (Canham and Burton, 1968; Secomb, 1991; Wang et al., 2022; Tomaiuolo et al., 2016).

As previously mentioned, the cohesion between the plasma membrane and the adjacent cytoskeleton in the RBCs from patients with HS is weakened. Abnormal cells undergo further damaged when passing through the spleen. When passing through the narrow IES, the membrane of these cells is forced to deform, which, due to its insufficient stability, results in the generation of microvesicles and the shedding of the lipid bilayer (Garcia-Herreros et al., 2022; Dao et al., 2021). This process gradually reduces the surface area of the RBCs that fosters their further splenic trapping and eventual destruction. The composition of microvesicles is dependent on the gene which mutation underlies the HS. In microvesicles from erythrocytes with mutated ankyrin or spectrins, Band 3 and actin are present. Microvesicles from erythrocytes with mutated Band 3 have a reduced amount of Band 3. Microvesicles of all types are almost devoid of hemoglobin, other cytoplasmic, and cytoskeletal proteins (Eber and Lux, 2004; Perrotta et al., 2008; Reliene et al., 2002; Bosman et al., 2012).

**FIGURE 6:**
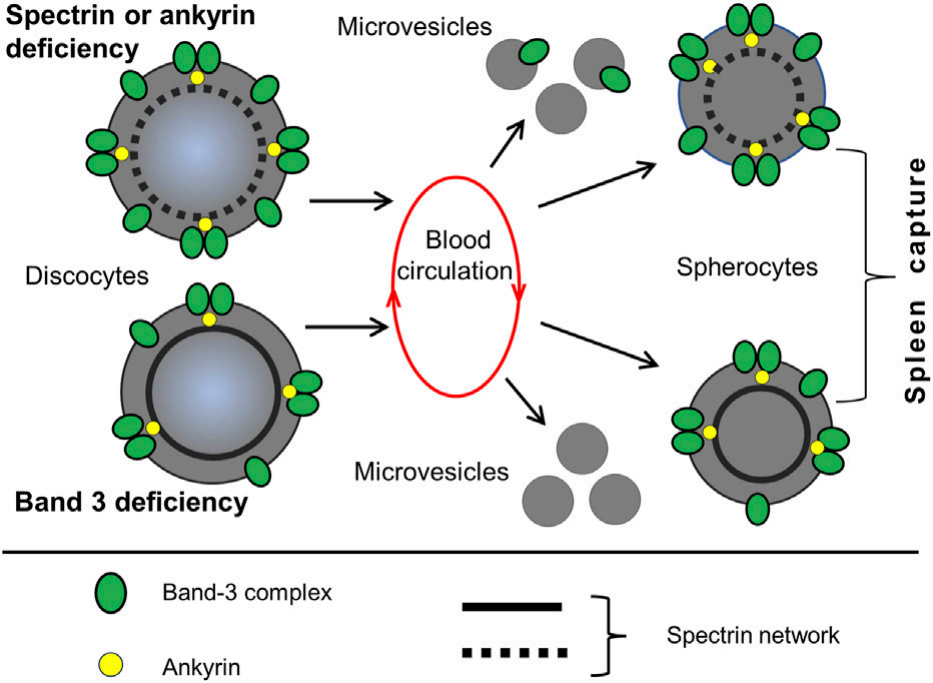
HS and the loss of membrane surface by vesiculation. A hallmark of HS is a decrease of RBC surface area and reduction in the cell surface to volume ratio Two different pathways lead to membrane shrinkage, which occurs during the passage of RBCs through the spleen. 1) Defects in spectrin, ankyrin, or protein 4.2 lead to reduced density of the membrane skeleton and destabilization of the lipid bilayer. This results in the generation of Band-3-containing microvesicles. 2) Defects in Band 3 lead to a weakening of the lipid bilayer connection with cytoskeleton, causing the formation of Band-3-depleted microvesicles. Both pathways lead to a reduction in the surface area of red cells and the formation of spherocytes, which have increased rigidity and decreased deformability. Spherocytes are subsequently retained in the spleen. This illustration is a merged figure adapted from both (Perrotta et al., 2008; Reliene et al., 2002).

With passage through the spleen, which is a repetitive event, RBCs can lose up to 20% of their surface area with a relatively small volume change. As a result, spherocytes cannot pass through the IES and remain in the red pulp, where they are destroyed by macrophages. The shedding of vesicles and reduction in membrane surface is occurs more easily in cells with spectrin mutations than in cells with Band 3 protein mutations. Due to membrane loss, spherocytes have an average surface area of 97 μm^2^ and a volume of 90 μm³, with an S/V ratio of approximately 1.1. The calculation shows that the minimum value for the surface area of a spherocyte with a volume of 90 μm³, required to escape spleen trapping, is 110 μm^2^. This means that the minimum S/V ratio is 1.2, which is slightly different from the experimental figure mentioned above (Vives Corrons et al., 2021; Li et al., 2018). As the surface area of biconcave red cells decreases, they gradually change their shape and transform firstly into concave stomatocytes, then spherocytes. This causes the cells to become stiff and lose elasticity. This is quantified by an increase in Young’s modulus, which for discocytes and spherocytes is equal to 26 ± 6.7 and 43 ± 6.2 Kpa, respectively (Figure 6) (Eber and Lux, 2004; Tomaiuolo, 2014).

Along with the spleen, damaged and, in particular, senescent RBCs are destroyed in the liver (so-called cell clearing process). Specific markers on the surface of erythrocytes serve as selection criteria (Slusarczyk and Mleczko-Sanecka, 2021). Oxidation and denaturation of hemoglobin contribute to the formation of clusters of Band 3 protein, which are recognized by naturally occurring antibodies (NAbs). These antibodies have a low affinity for Band 3, binding only if they are clustered together. Senescent red blood cells are recognized by macrophages and are phagocytized after binding of NAbs to Band 3 (Vives Corrons et al., 2021; Rifkind and Nagababu, 2013; Kay, 2005). In the liver, defective red blood cells are phagocytosed by specialized macrophages called Kupffer cells, which are similar to macrophages of the spleen parenchyma. In some physiological and pathological conditions, such as malaria infection, phosphatidylserine is exposed on the surface of RBCs. Kupfer cells have several phosphatidylserine receptors on their surface (TIM4, AXL, MERTK, STAB1; STAB2, CD36). It is worth noting that no increase in phosphatidylserine exposure on the outer leaflet of the plasma membrane was determined in RBCs from patients with HS. The function of transmembrane protein CD36, the Fc receptor, is to recognize Nabs attached to the clusters of Band 3. In addition, liver macrophages express SIRPα receptors that recognize the CD47 protein, abundant on the surface of young RBC. The binding of CD47 to SIRPα inhibits phagocytosis. As RBCs age, the conformation of CD47 changes, decreasing its affinity to SIRPα. The level of SIRPα expression in Kupfer cells is suppressed by pro-inflammatory cytokines and bacterial lipopolysaccharides (Slusarczyk and Mleczko-Sanecka, 2021; Kay, 2005; Föller and Lang, 2020; Borges and Sesti-Costa, 2022; de Jong et al., 1999).

## 7 Splenectomy

Splenectomy is a common treatment of patients with moderate to severe HS, mostly in childhood, if repetitive transfusions are indispensable. Only in very small number patients with extremely severe HS splenectomy leads to an incomplete improvement of their health. However, between 0.05 and 0.3 patients die from post-splenectomy sepsis every year out of every 100 patients who undergo the surgery. Therefore, the reasons for performing the surgery must be carefully considered. Due to the high risk of post-splenectomy infection in early childhood, it is recommended to delay total splenectomy until 6–9 years of age, if possible, and in any cases, it should not be performed before the age of 3 years. In life-threating cases partial (or subtotal) splenectomy may be used. Partial splenectomy is associated with a decreased risk of post-splenectomy sepsis. Several studies indicate that partial removal of 80%–90% of an enlarged reduces the rate of hemolysis and increases the lifespan of RBC while maintaining efficient phagocytic function of the spleen (Iolascon et al., 2017; Perrotta et al., 2008).

Additionally, it is important to differentiate HS from other types of anemia, such as hereditary stomatocytosis (both types, overhydrated and dehydrated) and hereditary xerocytosis. In case of dehydrated hereditary stomatocytosis, which is the most common form of the disease, splenectomy is contraindicated due to a high rate of death from thromboembolic events (Delaunay, 2007; Eber and Lux, 2004; Perrotta et al., 2008; van Vuren et al., 2019; Kalfa, 2021). Splenectomy is a risk factor for the development of vascular complications and pulmonary arterial hypertension, particularly in patients with various hemolytic disorders. The loss of splenic function is associated with an increased number of platelets and also enhances their activation inducing chronic microthrombosis in the pulmonary vessels. Additionally, splenectomy can provoke adhesion of RBCs to the endothelium, as the spleen, along with the liver, participates in removing senescent and damaged erythrocytes (Iolascon et al., 2017). Post-splenectomy predisposition to thrombosis and pulmonary hypertension are not unique to HS and hereditary stomatocytosis patients. Post-splenectomy thrombosis can develop in other hemolytic anemias like dyserythropoietic anemia, myeloproliferative disorder, and immune thrombocytopenia (Baldari et al., 2023; Iolascon et al., 2017; Boyle et al., 2013).

As we mentioned above, according to atomic force microscopy studies, spherocytes have shape that resembles oblate spheroids (or rotational ellipsoids) with semi-axes *a* and *b* approximately 2.7 μm and semi-axis *c* approximately 1.8 μm. This means that spherocytes appear to partly retain the ability to change shape. Splenectomy has a minimal effect on the morphological parameters of erythrocytes in patients with HS. The size of discocytes remains smaller compared to healthy control (Cloos et al., 2023). A study of spherocyte morphology using atomic force microscopy showed that after splenectomy, parameters such as the minimum and maximum height on the *Z*-axis of cells increased unexpectedly, becoming more different from those of healthy cells (Starodubtseva et al., 2019; Li et al., 2016).

After the removal of the spleen, its function to eliminate old and damaged RBC is completely transferred to the liver. Unlike the spleen, the liver does not verify the morphology of cells, but selects abnormal cells through recognition of specific markers, such as Nabs-Band 3 complexes and CD47. It was shown that the life span of erythrocytes was not increased in mice that had undergone splenectomy. The function of RBCs sequestration was adopted by the liver, which almost doubled this activity (Föller and Lang, 2020; Anosa, 1976).

After splenectomy, most physiological symptoms of HS return to normal. There is an increase in the number of RBCs and hemoglobin levels which results in a significant decrease in anemia and tissue hypoxia, accompanied by a large decrease in the percentage of reticulocytes and the level of erythropoietin in the blood, as well as a decrease in total bilirubin levels (Huisjes et al., 2020; Iolascon et al., 2017). Splenectomy helps to increase the lifespan of RBCs and prevents the rise in erythropoiesis caused by the anemic stress. As noted, along with quality control function, the recognition and removal of abnormal erythrocytes, the spleen plays a principal role in damaging of RBCs and generation of spherocytes. An increase in the lifespan of RBCs is displayed by an increase in the levels of glycated hemoglobin and deaminated isoform Band 4.1 (4.1a) detected by SDS-PAGE analysis of lysates of erythrocyte shadows. Both parameters increase during non-enzymatic modifications of hemoglobin and Band 4.1 protein in 1st order reactions and serve as indicators of the average age of RBC populations (Reliene et al., 2002; Cloos et al., 2023; Huisjes et al., 2020). According to the rate of glycated hemoglobin, splenectomy completely restores the lifespan of RBCs with all types of mutations (Huisjes et al., 2020). However, according to the rate of Band 4.1 deamination, splenectomy prolongs the survival of mature RBCs in spectrin/ankyrin and less so in those with Band 3 deficiency (Reliene et al., 2002).

After splenectomy, red cell size distribution width decreases to values seen in healthy individuals, indicating a reduction in the size and volume variability of RBCs. Additionally, the percentage of hyperchromic cells, mean corpuscular hemoglobin concentration as well as the density of RBC also return to normal levels, suggesting a decrease in hemoglobin denaturation and cell dehydration. In patients with HS, the effect of splenectomy on RBC vesiculation is not well understood. Direct measurements of membrane shedding by monitoring vesicles in blood plasma is challenging due to their fast elimination. However, a decrease in mean corpuscular hemoglobin concentration and hyperchromic cells after splenectomy could indicate that, under physiological conditions, the generation of RBC-derived vesicles is reduced after surgery, as elevation in mean corpuscular hemoglobin concentration is caused by the shedding of hemoglobin-free microvesicles and subsequent membrane loss (Berrevoets et al., 2021; Huisjes et al., 2020). As we mentioned earlier, membrane shedding mainly occurs in the spleen during the passage of RBCs through the IES. A substantial functional improvement in the RBC membrane status after splenectomy was detected using cell membrane stability test (CMST), an ektacytometry modification, that measures the RBC response to high shear stress. This finding suggests a partial restoration of the RBC ability to shed membrane, a characteristic of healthy cells that is reduced in matured spherocytes (Berrevoets et al., 2021; Cloos et al., 2023).

At the same time, according to ektacytometry test, splenectomy causes an insignificant improvement in reduced cell deformability, which is a marker of HS (parameters EI_max_, O_hyper_ and others determined by ektacytometry). Also, after splenectomy, the reduced intracellular concentration of K^+^ remains unaffected, as well as the low EMA binding and decreased osmotic stability according to OFT and cryohemolysis tests. The normalization of the parameter MCV (mean cell volume) is slight (Perrotta et al., 2008; Berrevoets et al., 2021; Cloos et al., 2023; Huisjes et al., 2020). It can be concluded that removal of the spleen does not correct the majority of genetically determined membrane abnormalities in erythrocytes in patients with HS (Table 2). After splenectomy, the transformation of discocytes into spherocytes still continues to occur, although at a reduced rate. In patients with mutations in the *SPTB* and *ANK1* genes, the proportion of spherocytes among RBCs decreases by approximately half after surgery. Specifically, this decrease is from 25% to 13% (Cloos et al., 2023). Additionally, the duration of life of RBCs increases by about 50% after splenectomy (Reliene et al., 2002). Therefore, splenectomy by increasing the lifespan of red cells, significantly reduces the severity of anemia and prevents cholelithiasis and hyperbilirubinaemia, improving the physiological signs of HS (Perrotta et al., 2008; Shah and Vega, 2004; Narla and Mohandas, 2017; Iolascon et al., 2017).

**TABLE 2:** Parameters characterizing the severity of HS and the effect of splenectomy.

Disease severity	MCHC	K^+^	Density	Reticulocytes	RDW	EI_max_	Vesiculation	HbA1c
Mild	↑	↓	↑	↑	N	↓	↑	↓
Moderate and severe	↑	↓↓	↑↑	↑↑	↑↑	↓↓	↑↑	↓↓
Splenectomized	↑	↓	N	N	N	↓	N	N

The ↑ symbol indicates an increase compared to healthy control groups; the ↓ symbol indicates a decrease. N indicates a normal value as in healthy control groups. Abbreviations in the Table: HbA1c, glycated hemoglobin; MCHC, means corpuscular hemoglobin concentration; RDW, red cell size distribution width; EI_max_, maximal elongation index obtained by ektacytometry, reflects the deformability of cells (Berrevoets et al., 2021; Huisjes et al., 2020).

## 8 Conclusion

The primary lesion in HS is the weakening of the attachment between the RBC plasma membrane and the cytoskeleton. This attachment is mediated by up to hundred proteins, but mutations in only a few of them (α- and β-spectrin, ankyrin-1, Band 3, and 4.2) lead to HS. Molecular defects can occur in most of the functional domains of these proteins and the clinical manifestations that they cause can vary greatly in severity. In most severe cases, splenectomy may be necessary to prevent RBC collapse caused by shedding of membranes during the passage of RBCs through the narrow slits of venous sinuses in the spleen. After splenectomy, the liver adopts the function of recognizing and eliminating senescent and abnormal RBCs. Unlike the spleen, which selects RBCs on the basis of their shape, liver macrophages identify abnormal red cells via their specific surface markers.

After splenectomy, the formation of spherocytes continues, although to a lesser degree than before surgery. It remains unclear whether the conversion of discocytes into spherocytes after splenectomy is a spontaneous process or the result of mechanical stress caused by cell passing through capillaries. Additionally, there is no complete understanding of the physicochemical and biochemical processes involved in the passage of spherocytes through capillaries with smaller diameter than the size of spherocytes.

In a course of maturation, erythrocytes undergo significant simplification, while still retaining a significant portion of their biochemical and regulatory systems that were present in their progenitor cells. These systems can be affected by pharmacological agents with the potential aim to correct the physiology of congenitally abnormal cells. The targets for these interventions can be pathways including ion channels, receptors of intercellular signaling molecules, protein kinases, protein phosphatases, and phosphodiesterases. Several pharmacological agents, such as pentoxifylline, mitapivat, verapamil, and imatinib, have earlier been tested to increase the deformability of RBCs in SCD and thalassemia (Noomuna et al., 2020; Cilek et al., 2024; Ugurel et al., 2019; Matte et al., 2023; Brun et al., 2021; Muravyov and Tikhomirova, 2013). Of them, mitapivat, a pyruvate kinase activator, was shown to alleviate anemia in a mouse model of human HS by reprograming RBC metabolism (Matte et al., 2023). Further studies are required to develop possible pharmacological approaches for the treatment of HS. Also, further studies are required to better understand the molecular mechanisms that underlie the remarkable ability of erythrocytes to undergo reversible deformation and withstand continuous mechanical and chemical stress.

## Abbreviations

AGLT, acidified glycerol lysis test; HS, hereditary spherocytosis; IES, interendothelial slit(s); Nabs, naturally occurring antibodies; OFT, osmotic fragility test; RBC, red blood cell; RhAG, Rh-associated glycoprotein; SCD, sickle cell disease.
